# The Effects of Interval Training and Continuous Training on Cardiopulmonary Fitness and Exercise Tolerance of Patients with Heart Failure—A Systematic Review and Meta-Analysis

**DOI:** 10.3390/ijerph18136761

**Published:** 2021-06-23

**Authors:** Daxin Li, Ping Chen, Junying Zhu

**Affiliations:** Department of Physical Education, Ocean University of China-Laoshan Campus, Qingdao 266100, China; lidaxin@stu.ouc.edu.cn (D.L.); qdchping@126.com (P.C.)

**Keywords:** interval training, continuous training, heart failure, meta-analysis

## Abstract

Purpose: To investigate the effects of interval training (IT) as compared with continuous training (CT) on cardiorespiratory fitness and exercise tolerance of patients with heart failure (HF), with the aim to provide reasonable exercise prescriptions for patients with HF. Methods: Through searching electronic databases, randomized controlled studies were collected. The included studies were evaluated for methodological quality using the Cochrane risk of bias assessment tool, and statistical analyses were carried out using Review Manager 5.3 and Stata MP 15.1 software. Results: A total of seventeen randomized controlled trials (i.e., studies) with 617 patients were included. The meta-analysis showed that IT can improve a patient’s peak oxygen uptake (VO2peak) (MD = 2.08, 95% CI 1.16 to 2.99, *p* < 0.00001), left ventricular ejection fraction (LVEF) (MD =1.32, 95% CI 0.60 to 2.03, *p* = 0.0003), and 6-minute walk distance (6MWD) (MD = 25.67, 95% CI 12.87 to 38.47, *p* < 0.0001) as compared with CT. However, for respiratory exchange ratio (RER) (MD = 0.00, 95% CI −0.02 to 0.03, *p* = 0.81), CO_2_ ventilation equivalent slope (VE/VCO2 slope) (SMD = 0.04, 95% CI −0.23 to 0.31, *p* = 0.75), and resting heart rate (HRrest) (MD = 0.15, 95% CI −3.00 to 3.29, *p* = 0.93) there were no statistical significance. Conclusions: The evidence shows that IT is better than CT for improving the cardiorespiratory fitness and exercise tolerance of patients with HF. Moreover, an intensity of 60–80% peak heart rate of IT is the optimal choice for patients. It is hoped that, in the future, more well-designed studies would further expand the meta-analysis results.

## 1. Introduction

Heart failure (HF) is a common disease with an increasing prevalence worldwide and it is characterized by a low five-year survival of 35–55% [1], which affects cardiac function, exercise tolerance, and the daily life of patients [2,3]. Cardiac rehabilitation is defined as a set of activities that aims to provide patients with heart disease with the best physical, mental, and social conditions, therefore, reducing the risk of death and acute events related to their illness [4]. Previous studies have demonstrated that cardiac rehabilitation with physical exercise was beneficial to physical fitness, cardiac function, and quality of life in HF patients [5,6]. At present, various exercise programs are widely applied to cardiac rehabilitation, in which continuous training (CT) and interval training (IT) are the main forms of exercise [7,8]. CT is defined as continuous training with low and moderate intensity exercises that are performed for more than 20 min without resting intervals. IT is characterized by relatively high-intensity repetitions of physical activity with periods of rest for recovery [9]. It has been widely demonstrated that CT improves aerobic capacity, skeletal muscle function, and quality of life. In addition, it can change peripheral blood flow and decrease mortality rate [10,11,12]. However, CT as an exercise program can be tedious for the patients, which results in the exercise effect being unsustainable [13]. Therefore, IT has been increasingly used in cardiac rehabilitation for HF patients [7,14]. IT leads to greater improvements in aerobic capacity, left ventricular function, endothelial function, and quality of life [15,16]. In addition, IT for patients with HF appears to be more effective than CT for improving functional capacity [17]. However, there continues to be disagreement on whether or not IT and CT can significantly improve the cardiac function and functional capacity of patients with cardiovascular disease; the effectiveness between the two exercise programs is similar and it cannot be distinguished which exercise program is better [18,19].

Some previous studies have shown that the two exercise programs were effective in cardiac rehabilitation of HF patients [4,20]. However, due to differences in subjects and intervention programs, the conclusions were still controversial. Neil compared the effect of IT and CT in patients with HF, and showed that IT elicited superior improvements in peak oxygen uptake (VO2peak) and CO_2_ ventilation equivalent slope (VE/VCO2 slope) as compared with CT in HF patients [20]. VO2peak has been considered to be the best predictor of survival in cardiovascular diseases and it has been used in many previous studies to measure patients’ cardiorespiratory fitness [17,18,19,20,21]. The VE/VCO2 slope is inversely related to cardiac output at peak exercise and is at least partly explained by a decrease in pulmonary perfusion [22]. This prognostic parameter related to cardiac function has been chosen consistently in HF patients [18,20,23,24,25,26,27]. Bruna (2019) suggested that high intensity interval training was more effective than moderate continuous interval training for improving VO2peak, while the effect was not significant for improving left ventricular ejection fraction (LVEF) between the two exercise programs [4]. LVEF is a sensitive index that directly reflects the left ventricular ejection efficiency and indirectly reflects myocardial contractility [19,28]. Because of its close association with HF, the prognostic value that the LVEF consistently demonstrates is not surprising [19,20,23,26,27,29,30]. The number of included studies was inadequate (only five studies) in the above two studies, which were not enough for them to state whether IT was superior to CT. Mansueto (2018) suggested that high intensity interval training was superior to moderate continuous interval training for improving VO2peak in HF patients with reduced ejection fraction but the superiority disappeared when they performed a subanalysis [31]. The aim of this systematic literature review with meta-analysis was to synthesize the most up-to-date evidence to explore the effects of IT and CT on cardiorespiratory fitness and exercise tolerance of patients with HF. The specific objectives were:

1. To compare the effects of IT and CT on cardiorespiratory fitness and exercise tolerance of patients with HF (subanalysis with different durations and isocaloric consumption).
2. To compare difference high or moderate intensities of IT on cardiorespiratory fitness and exercise tolerance, to provide an optimal exercise prescription for patients with HF.
3. To collect rehabilitation recommendations for future research on this topic.

## 2. Methods

### 2.1. Literature Search

A systematic literature review was conducted in Pubmed, Embase, Cochrane library, Web of Science, China Biomedical Literature Database, China National Knowledge Infrastructure, VIP Database, and Wanfang Data. The randomized controlled trials were collected between the earliest available date and April 2021 using the following terms: (high intensity interval training OR high-intensity intermittent exercise OR sprint interval training OR aerobic interval training OR interval training) AND (heart failure OR congestive heart failure OR myocardial failure OR heart decompensation OR cardiac insufficiency). In addition, the references of articles included in other systematic reviews with meta-analyses were searched to identify other possible eligible studies.

### 2.2. Study Selection

The inclusion criteria for this meta-analysis were full-text research articles published in peer-reviewed academic journals in Chinese or English language. The exclusion criteria were: (1) patients with unstable HF, (2) non-randomized controlled trials, (3) outcome measurements that did not meet the requirements, (4) a significant difference between the baseline values of the two groups (*p* < 0.05), (5) patients who had no medical supervision during the exercise intervention.

Two researchers independently screened the literature by reading the titles and abstracts and excluded irrelevant studies. Then, they independently collected and downloaded the studies that met the standards and excluded the unqualified studies by reading the full text. Differences in the assessment of study eligibility were resolved by discussion.

### 2.3. Measured Outcomes

The primary outcome measurement was changes in VO2peak (mL/kg/min). Secondary outcomes included cardiorespiratory fitness parameters (i.e., respiratory exchange ratio (RER), LVEF, and resting heart rate (HRrest)) and exercise tolerance parameters (i.e., VE/VCO2 and 6-minute walk distance (6MWD)).

### 2.4. Data Extraction and Analysis

All data were independently extracted by an investigator and checked for accuracy by another reviewer. The collected data included authors’ names, year of publication, country in which the study was conducted, characteristics of participants, intervention description, outcome, and quality assessment.

### 2.5. Quality Assessment

The study quality was assessed by two authors using *Cochrane Handbook for Systematic Reviews of Interventions* 5.0.1 which included selection bias, performance bias, detection bias, attrition bias, reporting bias, and other biases. Disagreements were resolved by consensus [32].

### 2.6. Statistical Analysis

Statistical analyses were performed using Review Manager 5.3 (Nordic Cochrane Centre, Copenhagen, Denmark) and Stata MP 15.0 (StataCorp, Pyrmont, Australia). Effect sizes for continuous variables were expressed as either mean difference (MD) or standardized mean difference (SMD), each with 95% confidence interval (95% CI). The heterogeneity among studies was examined with Cochrane’s Q and I^2^ statistics, in which values greater than 50% indicated significant heterogeneity and random-effects model was chosen [33]. The overall effects were considered to be significant when *p*-values (*p*) were ≤0.05. A sensitivity analysis with one-by-one removal of studies was conducted to investigate possible effects of each study on heterogeneity and overall effect. Finally, Egger’s regression model was used to assess publication bias.

## 3. Results

### 3.1. Identified Studies

**Figure 1:**
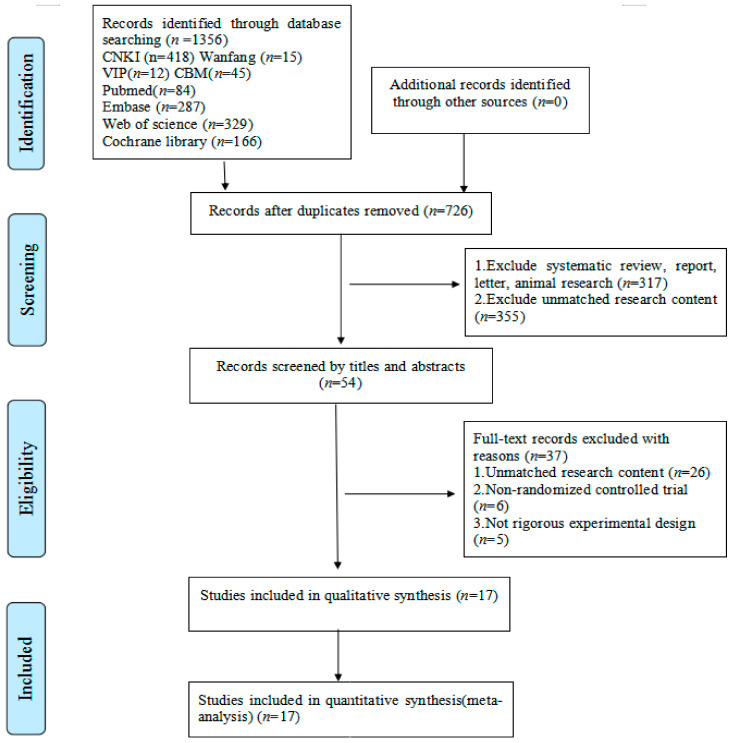
Flow diagram of literature selection.

The initial research resulted in 1356 references. After duplicates were removed, the titles and abstracts of 726 studies were reviewed. Following a screening of potential studies, 672 studies were excluded, and 54 studies were retrieved in full text, 37 studies of which did not match the eligibility criteria. The final seventeen studies were included in the meta-analysis (Figure 1).

### 3.2. Study Characteristics

**Figure 2:**
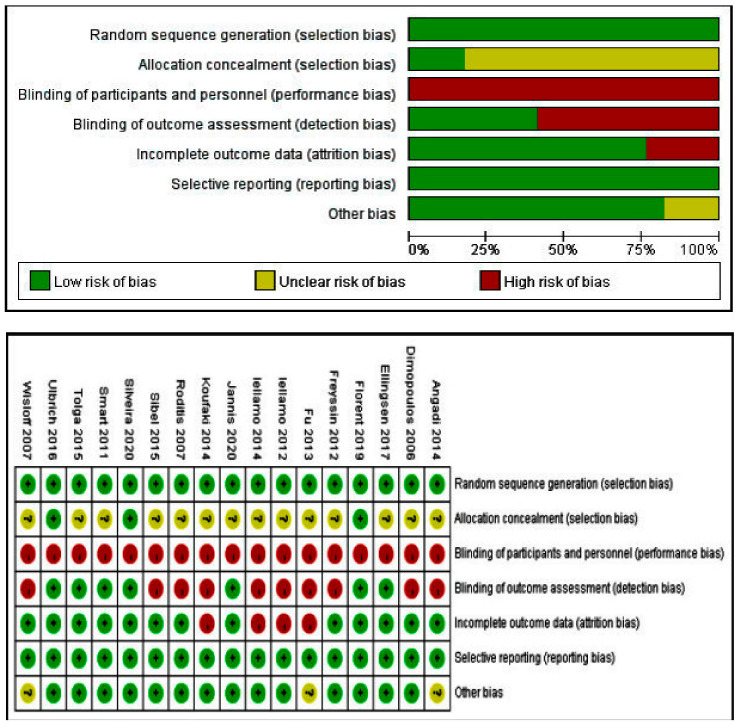
Analysis of the risk of bias in accordance with the Cochrane collaboration guidelines.

The characteristics of included studies are shown in Table 1 and Table 2 and the methodological quality of each study is shown in Figure 2. The seventeen studies involved a total of 617 patients (316 IT and 301 CT) with HF [17,18,19,20,23,24,25,26,27,29,30,34,35,36,37,38,39]. Among these studies, two studies each were conducted in Brazil [19,27], France [17,26], Greece [40,41], Italy [18,24]. Norway [34,37] and Turkey [25,36], one study each was conducted in the America [29], Australia [20], Bulgaria [27], China/Taiwan [35] and England [29]. Intervention duration ranged from 3 to 24 weeks with a frequency of exercise training ranging from 2 to 5 days per week.

Randomization was adopted in each study, of which nine studies described specific randomization [20,25,26,27,29,30,34,35,38]. Because the patients were older, which could lead to adverse accidents, all patients needed to have signed informed consent forms and only seven studies implemented blinding and all of them were blind to the assessor [19,20,27,30,37,38,39]. Four studies reported the dropout of patients and the reasons for dropout were indicated in the study [18,23,24,25].

### 3.3. Effects of the Intervention

#### 3.3.1. VO2peak

**Figure 3:**
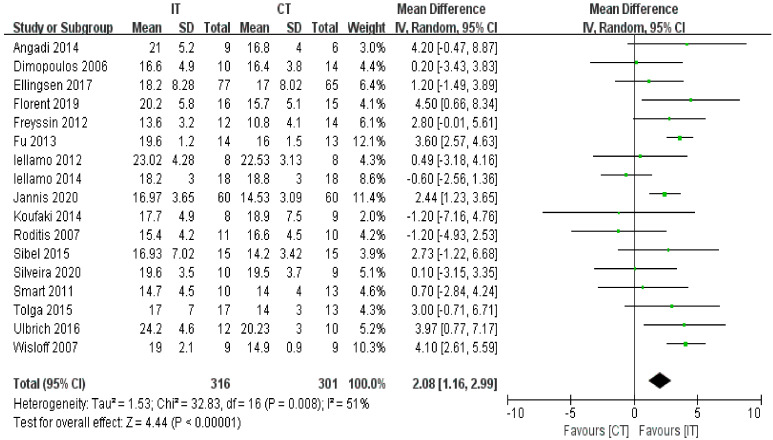
Forest plot: Effects of VO2peak.

VO2peak was reported by seventeen studies including 617 participants with HF. The aggregate results of these studies showed that IT was associated with a significantly improved VO2peak (random effects model, MD = 2.08, 95% CI 1.16 to 2.99, *p* < 0.00001) (Figure 3). The test for heterogeneity was significant (*p* = 0.008 and I^2^ = 51%). Subgroup analyses based on intervention duration, exercise intensity of IT, and isocaloric consumption were performed. The results of subgroup analyses (Table 3) showed that intervention duration, exercise intensity of IT, and isocaloric consumption were not the potential factors that led to heterogeneity. Sensitivity analyses were conducted to explore potential sources of heterogeneity, exclusion of individual studies did not substantially alter heterogeneity.

#### 3.3.2. RER

**Figure 4:**
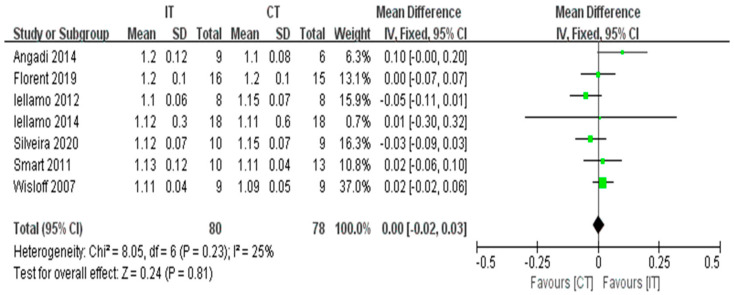
Forest plot: Effects of the RER.

Seven studies with a total of 158 participants reported no significant difference in the RER between IT and CT (fixed-effects model, MD = 0.00, 95% CI −0.02 to 0.03, *p* = 0.81) (Figure 4). The test for heterogeneity was not significant (*p* = 0.23 and I^2^ = 25%).

#### 3.3.3. VE/VCO2 Slope

**Figure 5:**
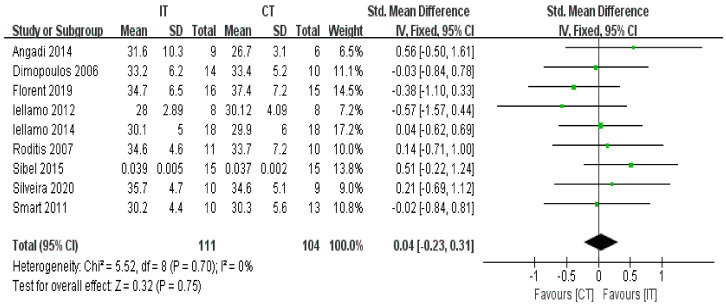
Forest plot: Effects of VE/VCO2 slope.

Nine studies with a total of 215 participants reported no difference in the VE/VCO2 slope between IT and CT (fixed-effects model, SMD = 0.04, 95% CI −0.23 to 0.31, *p* = 0.75) (Figure 5). The test for heterogeneity was not significant (*p* = 0.70 and I^2^ = 0%).

#### 3.3.4. LVEF

**Figure 6:**
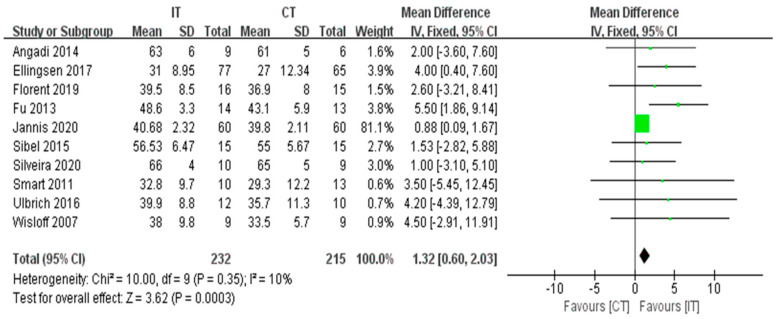
Forest plot: Effects of LVEF.

The LVEF was reported by ten studies that included a total of 447 participants with HF Figure 6). The meta-analysis showed a significant improvement for participants in the IT group as compared with the CT group (fixed-effects model, MD = 1.32, 95% CI 0.60 to 2.03, *p* = 0.0003) (Figure 6). The test for heterogeneity was not significant (*p* = 0.35 and I^2^ = 10%).

#### 3.3.5. HRrest

**Figure 7:**
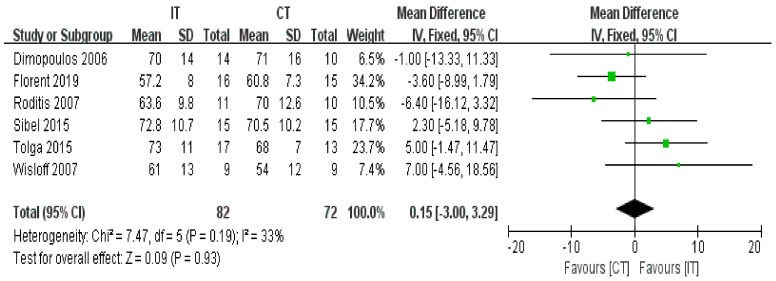
Forest plot: Effects of HRrest.

Six studies with a total of 154 participants reported no difference in HRrest between IT and CT (fixed-effects model, MD = 0.15, 95% CI −3.00 to 3.29, *p* = 0.93) (Figure 7). The test for heterogeneity was not significant (*p* = 0.19 and I^2^ = 33%).

#### 3.3.6. MWD

**Figure 8:**
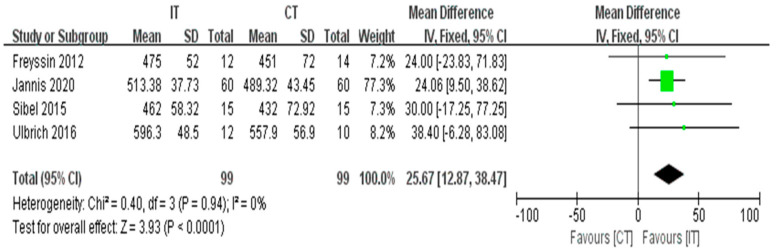
Forest plot: Effects of 6MWD.

Four studies with a total of 198 participants reported a significant difference in 6MWD between IT and CT (fixed-effects model, MD = 25.67, 95% CI 12.87 to 38.47, *p* < 0.0001) (Figure 8). The test for heterogeneity was not significant (*p* = 0.94 and I^2^ = 0%).

#### 3.3.7. Publication Bias

**Figure 9:**
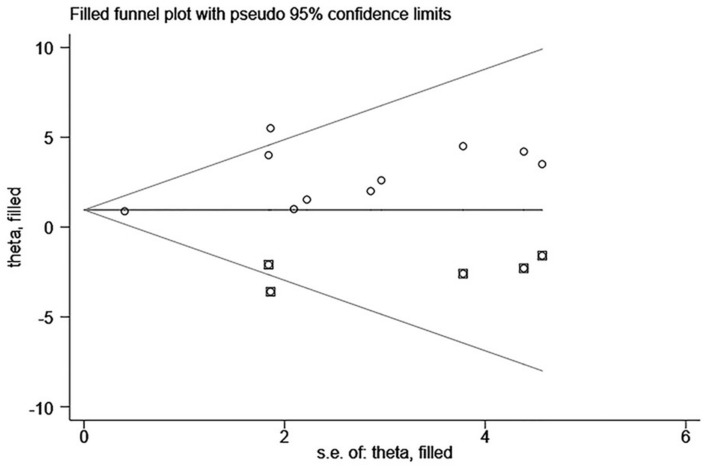
O: previous studies; **□**: filled studies. A funnel plot with trim and fill for the effect size of LVEF.

Egger’s test was applied for the six outcomes (Table 4). There were no significant publication biases for VO2peak, RER, VE/VCO2 slope, HRrest, and 6MWD. However, there was publication bias for LVEF (asymmetry test, *p* = 0.022). Therefore, the trim-and-fill method which conservatively imputes hypothetical negative unpublished studies to mirror the positive studies that cause funnel plot asymmetry was performed. The imputed studies produced a symmetrical funnel plot (Figure 9). Combined with the funnel chart, the five studies need to be included, in the future, to ensure the symmetry of the funnel chart and eliminate publication bias.

## 4. Discussion

This systematic literature review with meta-analysis suggests that IT elicits greater improvements in VO2peak, LVEF, and 6MWD than CT, which is similar to previous meta-analyses comparing IT with CT in HF [4,42] and coronary heart disease patients [43,44]. The strengths of this study as compared with previous studies is that more studies were retrieved to compare the effects on cardiorespiratory fitness and exercise tolerance in HF patients between IT and CT. In addition, several indispensable outcomes for HF patients were adopted to measure the effects between the two exercise programs, and therefore provided enough basis for cardiac rehabilitation.

The VO2peak is considered to be the best predictor of survival in cardiovascular diseases [45,46]. Previous studies have indicated that a peak aerobic power ≤10 mL/kg/min is a strong predictor of a poor prognosis in patients with HF [47,48]. The meta-analysis showed that IT significantly improved VO2peak of 2.08 mL/kg/min in patients with HF than CT. In addition, the results of the subgroup analyses suggested that IT as compared with CT was more significant for improving patients’ VO2peak with “intervention duration <12 weeks” than “intervention duration ≥12 weeks”. Meanwhile, the intensity of 60–80% HRpeak can gain better exercise effects than the intensity of 80–100% HRpeak for HF patients. The reason why a lower intensity gained a better effect may be that maximal intensity of IT has a deeper impact on patients’ hearts than a relatively lower intensity, which may not be beneficial to recovery. Previous clinical studies have shown that every 1 mL/kg/min increment in VO2peak leads to the mortality of male and female patients with cardiovascular diseases reducing by 16% and 14%, respectively [21]. The mechanism of IT improving VO2peak may be reflected in the following aspects: (1) the intensity of IT is relatively higher than CT, which may result in an increase in plasma volume and erythrocyte volume [49,50]. (2) IT improves venous drainage and increases stroke output as well as decreases the resistance of blood flow [51]. (3) IT can increase activation of peroxisome proliferator-activated receptor-γ coactivator (PGC-1a), which accelerates the mitochondrial biosynthesis process, which is essential to enhance the metabolism ability of skeletal muscle. Mitochondrial function is associated with aerobic physical fitness and plays an important pathophysiological role in cardiac patients [43,52]. Some previous studies have explored the potential physiological mechanism of IT for improving patients’ cardiorespiratory fitness, but there was still no clear explanation. It may be influenced by intervention duration, exercise intensity, and individual physical capacity. Therefore, the physiological mechanism of IT for improving cardiorespiratory fitness needs further exploration.

The LVEF is a sensitive index that reflects the function of the left ventricular pump. It is more sensitive and reliable than stroke volume and cardiac index. It directly reflects the left ventricular ejection efficiency and indirectly reflects myocardial contractility [28]. The meta-analysis suggested that there was a significant difference in the LVEF between IT and CT (MD = 1.32, 95% CI 0.60 to 2.03, *p* = 0.0003). The mechanisms responsible for an increment in LVEF may be the following: (1) A higher exercise heart rate during IT increases the magnitude of the post-exercise alteration in left ventricular diastolic filling [53]. (2) Potential mechanisms responsible for altered left ventricular relaxation, in addition to prolonged elevated heart rate, include downregulation of cardiac β-adrenoceptors mediated by elevated catecholamines during exercise. In fact, circulating catecholamines are responsible for maintaining tachycardia during exercise [54]. (3) Exercise training leads to a partial correction of peripheral endothelial dysfunction in patients with HF [55].

The 6MWD is an indicator of the ability to perform daily life activities, which measures exercise tolerance. Improvement in the 6MWD has also been equated with improved quality of life in patients [56]. The meta-analysis suggested that IT significantly increased 6MWD more than CT in HF patients (MD = 25.67, 95% CI 12.87 to 38.47, *p* < 0.0001). The mechanism of IT responsible for increased 6MWD is that a high-intensity IT effort culminating near VO2max, requires that mitochondrial oxidative phosphorylation is fueled by carbohydrate substrates and operates at or near maximal capacity for several consecutive minutes. This type of effort might also represent a greater metabolic challenge for the mitochondria than CT, during which anaerobic metabolism (glycolysis and phosphocreatine) contributes significantly to ATP production [57]. Finally, the acute effects on mitochondrial respiratory function of a relatively high-intensity IT that ultimately yields VO2max and elicits improvements in muscle aerobic capacity [52].

The RER is the ratio of carbon dioxide emission to oxygen uptake. A value of RER equal to at least 1.0 is commonly used to describe adequate effort and motivation in HF patients [58]. The result suggested that there was no significance in RER between IT and CT (MD = 0.00, 95% CI −0.02 to 0.03, *p* = 0.81). The VE/VCO2 slope is an important indicator reflecting exercise tolerance, and it is also an important predictor of death in patients with HF [59]. Risk of mortality is thought to increase when the value of the VE/VCO2 slope is greater than 34 [40]. The non-significance of our result (SMD = 0.04, 95% CI −0.23 to 0.31, *p* = 0.75) between IT and CT is in agreement with previous studies [43,44]. HRrest is a useful clinical marker for cardiovascular disease assessment. Previous studies have shown that for every 10 beats per minute (bpm) increment in HRrest, there is a 14% increased risk for a clinical cardiovascular disease event [41]. The meta-analysis result showed that there was no significance in HRrest between the two exercise programs (MD = 0.15, 95% CI −3.00 to 3.29, *p* = 0.93). These outcomes still need to be further elucidated in large and well-designed studies.

There are some limitations to the meta-analysis as follows: (1) There are no previous studies that have explored the impact of different intensities of IT on HF patients, which makes the division of intensity difficult. In addition, in all the included studies, all patients were in the New York Heart Association (NYHA) functional class I–III, but there was no literature to provide detailed class information. In the future, different intensities of IT could be classified to investigate which one is the optimal intensity for HF patients with different NYHA classes. (2) There is significant heterogeneity with respect to the outcome of VO2peak. Although various subgroups (i.e., exercise duration, exercise intensity of IT, and isocaloric consumption) were performed to explore heterogeneity, unwanted heterogeneity was still obvious, and the relatively small number of studies included in each subgroup could not effectively account for the heterogeneity underlying the various studies. (3) This meta-analysis is not registered and some outcomes are based on small sample sizes, which may affect the stability of the results. In addition, our results may be affected by publication bias. It is hoped that, in the future, more well-designed studies would further expand the meta-analysis results.

## 5. Conclusions

The evidence shows that interval training is better than continuous training for improving cardiorespiratory fitness and exercise tolerance of patients with heart failure. Moreover, the intensity of 60–80% peak heart rate of interval training is the optimal choice for patients. It is hoped that, in the future, more well-designed studies would further expand the meta-analysis results.

## Figures and Tables

**Table 1:** Characteristics of the studies included in the meta-analysis.

Study	Country	Characteristics of Patients				Outcome	Quality Assessment
		Sample Size (IT/CT)	Gender (M/F)	Age (years) (Mean ± SD)	Diagnosis Standard of HF		
Dimopoulos 2006 [34]	Greece	24 (14/10)	IT (9/1) CT (14/0)	IT (59.2 ± 12.2) CT (61.5 ± 7.1)	HFrEF HFmrEF HFpEF	VO2peak, VE/VCO2 Slope, HRrest	4
Roditis 2007 [35]	Greece	21 (11/10)	IT (10/1) CT (9/1)	IT (63 ± 2) CT (61 ± 3)	HFrEF HFmrEF	VO2peak, VE/VCO2 Slope, HRrest	4
Wisloff 2007 [29]	Norway	18 (9/9)	IT (7/2) CT (7/2)	IT (76.5 ± 9) CT (74.4 ± 12)	HFrEF	VO2peak, RER, LVEF, HRrest	3
Smart 2011 [20]	Australia	23 (10/13)	IT (8/2) CT (13/0)	IT (59.1 ± 11) CT (62.9 ± 9.3)	HFrEF	VO2peak, RER, VE/VCO2 slope, LVEF	5
Freyssin 2012 [17]	France	26 (12/14)	IT (6/6) CT (7/7)	IT (54 ± 9) CT(55 ± 12)	HFrEF	VO2peak, 6WMT	4
Iellamo 2012 [18]	Italy	16 (8/8) dropout 20%	NI	IT(62.2 ± 8) CT (62.6 ± 9)	HFrEF	VO2peak, RER, VE/VCO2 slope	3
Fu 2013 [23]	Taiwan	30 (15/15) dropout 10%	IT (10/5) CT (9/6)	IT (67.5 ± 1.8) CT (66.3 ± 2.1)	HFrEF HFmrEF	VO2peak, LVEF	2
Koufaki 2014 [24]	England	33 (16/17) dropout 48%	IT (14/2) CT (13/4)	IT (59.8 ± 7.4) CT (59.7 ± 10.8)	HFrEF HFmrEF	VO2peak	3
Angadi 2014 [36]	America	15 (9/6)	IT (8/1) CT (4/2)	IT (69 ± 6.1) CT (71.5 ± 11.7)	HFpEF	VO2peak, RER, VE/VCO2 slope, LVEF	3
Iellamo 2014 [25]	Italy	36 (18/18) dropout 8%	IT (16/2) CT (15/3)	IT (67.2 ± 6) CT (68.4 ± 8)	HFrEF	VO2peak, RER, VE/VCO2 slope	3
Tolga 2015 [37]	Turkey	30 (17/13)	IT (13/4) CT (13/0)	IT (63.7 ± 8.8) CT (59.6 ± 6.8)	HFrEF HFmrEF	VO2peak, HRrest	5
Sibel 2015 [26]	Turkey	30 (15/15)	IT (13/2) CT (13/2)	IT (63.7 ± 8.8) CT (59.6 ± 6.9)	HFrEF HFmrEF HFpEF	VO2peak, VE/VCO2 slope, LVEF, HRrest, 6WMT	4
Ulbrich 2016 [19]	Brazil	22 (12/10)	IT (12/0) CT (10/0)	IT (53.15 ± 7) CT (54.02 ± 9.9)	HFrEF HFmrEF	VO2peak, LVEF, HRrest, 6WMT	6
Ellingsen 2017 [30]	Norway	142 (77/65)	IT (59/18) CT (53/12)	IT (63 ± 22.4) CT (61.5 ± 14.4)	HFrEF	VO2peak, LVEF	5
Florent 2019 [27]	France	31 (16/15)	IT (11/5) CT (11/4)	IT (59 ± 13) CT (59.5 ± 12)	HFrEF HFmrEF	VO2peak, RER, VE/VCO2 slope, LVEF, HRrest	6
Jannis 2020 [38]	Bulgaria	120 (60/60)	IT (35/25) CT (35/25)	IT (63.7 ± 6.7) CT (63.8 ± 6.7)	HFrEF	VO2peak, LVEF, 6WMT	5
Silveira 2020 [39]	Brazil	19 (10/9)	IT (3/7) CT (4/5)	IT (60 ± 10) CT (60 ± 9)	HFpEF	VO2peak, RER, VE/VCO2 slope, LVEF	6

**Table 2:** Characteristics of the studies included in the meta-analysis (intervention program).

Study	Intervention			
	Mode	Duration	Exercise Program	
			IT	CT
Dimopoulos 2006 [34]	Cycle ergometer	12 weeks, 3 d/week	Total: 40 min ① 40 × 30 s interval (100–120% WR peak) ② 40 × 30 s recovery	Total: 40 min 40 min cycling (50–70% WR peak)
Roditis 2007 [35]	Cycle ergometer	12 weeks, 3 d/week	Total: 40 min ① 40 × 30 s interval (100–120% WR peak) ② 40 × 30 s recovery	Total: 40 min 40 min cycling (50–60% WR peak)
Wisloff 2007 [29]	Treadmill	12 weeks, 3 d/week	Total: 38 min ① 10 min warm-up (60–70% HRpeak) ② 4 × 4 min interval (90–95% HRmax) ③ 3 × 3 min recovery (50–70% HRmax) ④ 3 min cool-down	Total: 47 min 47 min running (70–75% HRmax)
Smart 2011 [20]	Cycle ergometer	16 weeks, 3 d/week	Total: 60 min ① 30 × 60 s interval (70% VO2peak) ②3 0 × 60 s recovery	Total: 30 min 30 min cycling (70% VO2peak)
Freyssin 2012 [17]	Cycle ergometer/Treadmill	8 weeks, 5 d/week	Total: 74 min ① 10 min warm-up (5 W) ② (12 repetitions of 30 s of exercise and 60 s of recovery)*3 (50–80 W), separated by 5 min recovery	Total: 60 min ① 10 min warm-up ② 45 min running/cycling (HR_VT1_) ③ 5 min cool-down
Iellamo 2012 [18]	Treadmill	12 weeks, 2 d/1–3 weeks, 3 d/4–6 weeks, 4 d/7–9 weeks, 5 d/10–12 weeks	Total: 37 min ① 9 min warm-up ② 4 × 4 min interval (75–80% HRR) ③ 4 × 3 min recovery (45–50% HRR)	Total: 30–45 min 30–45 min running (45–60% HRR)
Fu 2013 [23]	Cycle ergometer	12 weeks, 3 d/week	Total: 60 min ① 30 × 60 s interval (60–70% VO2peak) ② 30 × 60 s recovery	Total: 30 min 30 min cycling (60–70% VO2peak)
Koufaki 2014 [24]	Cycle ergometer	24 weeks, 3 d/week	Total: 30 min (30 s × 10 interval (100% WR peak) 60 s × 10 recovery (20–30% WR peak) × 2	Total: 40 min 40 min cycling (40–60% VO2peak)
Angadi 2014 [36]	Treadmill	4 weeks, 3 d/week	Total: 31–43 min ① 10 min warm-up (50% HR peak) ② 4 × 2–4 min interval (80–90% HRpeak) ③ 4 × 2–3 min recovery (50% HR peak) ④ 5 min cool-down (50% HR peak)	Total: 30–45 min ① 10 min warm-up (50% HRpeak) ② 15–30 min running (60–70% HR peak) ③ 5 min cool-down (50% HRpeak)
Iellamo 2014 [25]	Treadmill	12 weeks, 3 d/week	Total: 48 min ① 10 min warm-up ② 4 × 4 min interval (75–80% HRR) ③ 4 × 3 min recovery (45–50% HRR) ④ 10 min cool-down	Total: 55–60 min ① 10 min warm-up ② 30–45 min running (45–60% HRR) ③ 10 min cool-down
Tolga 2015 [37]	Cycle ergometer	12 weeks, 3 d/week	① 5 min warm-up ② 30 s interval (50–75% HRR) with 30 s recovery (50–75% HRR) ③ 5 min cool-down	Total: 40 min ① 5 min warm-up ② 30 min cycling (50–75% HRR) ③ 5 min cool-down
Sibel 2015 [26]	Cycle ergometer	10 weeks, 3 d/week	Total: 35 min ① 10 min warm-up/cool-down (20 W) ② 17 × 60 s interval (50–75% VO2peak) ③ 17 × 30 s recovery (30 W)	Total: 35 min ① 10 min warm-up/cool-down (20 W) ② 25 min cycling (50–75% VO2peak)
Ulbrich 2016 [19]	Treadmill	12 weeks, 3 d/week	Total: 36–51 min ① 7–10 min warm-up (70% HR peak) ② 4–6 × 3 min interval (95% HR peak) ③ 4–6 × 3 min recovery (70% HRpeak) ④ 5 min cool-down (50% VO2peak)	Total: 42–45 min ① 7–10 min warm-up (70% HRpeak) ② 30 min Running (75% HRpeak) ③ 5 min cool-down (50% VO2peak)
Ellingsen 2017 [30]	Cycle ergometer/treadmill	12 weeks, 3 d/week	Total: 38 min ① 5 min warm-up ② 4 × 4 min interval (90–95% HRpeak) ③ 4 × 3 min recovery ④ 5 min cool-down	Total: 47 min 47 min cycling or running (60–70% HRpeak)
Florent 2019 [27]	Cycle ergometer	3 weeks, 5 d/week	Total: 30 min ① 5 min warm-up (30% WR peak) ② 2 × (30 s interval following 30 s recovery × 8)(100% WR peak), seperated by 4 min recovery ③ 5 min cool-down (30% WR peak)	Total: 40 min ① 5 min warm-up (30% WR peak) ② 30 min cycling (60% WR peak) ③ 5 min cool-down (30% WRpeak)
Jannis 2020 [38]	Cycle ergometer	12 weeks, 2 d/week	Total: 40 min ① Warm-up ② 3 bouts of interval (90% HRpeak) ③ 2 bouts of recovery (70% HRpeak) ④ Cool-down	Total: 40 min 40 min cycling (70% HRpeak)
Silveira 2020 [39]	Treadmill	12 weeks, 3 d/week	Total: 38 min ① 10 min warm-up ② 4 × 4 min interval (85–95% HRpeak) ③ 3 × 3 min recovery ④ 3 min cool-down	Total: 47 min 47 min running (60–70% HRpeak)

Abbreviations: IT, interval training; CT, continuous training; M/F, male/female; HRpeak, heart rate peak; HRmax, maximal heart rate; VO2peak, peak oxygen uptake; VO2res, reserve oxygen uptake; HRres/HRR, reserve heart rate; HR_VT1_, the first ventilatory threshold; WRpeak, peak work rate; NI, not informed; HFrEF, heart failure with reduced ejection fraction; HFmrEF, heart failure with mid-range ejection fraction; HFpEF, heart failure with preserved ejection fraction.

**Table 3:** Subgroup analyses of effects of IT vs. CT on VO2peak in HF patients.

Outcome	Subgroup	Potential Factors	Included Studies	Sample Size	95% Confidence Intervals	Heterogeneity	*p*-Value
VO_2_ peak	Intervention duration	Duration < 12 weeks	4	102	3.38 (1.56, 5.19)	I^2^ = 0% *p* = 0.87	*p* = 0.0003
		Duration ≥ 12 weeks	13	515	1.73 (0.65, 2.82)	I^2^ = 62% *p* = 0.002	*p* = 0.002
	Exercise intensity of IT	Intensity of 60–80% HRpeak	5	136	3.26 (2.38, 4.15)	I^2^ = 0% *p* = 0.62	*p* < 0.00001
		Intensity of 80–100% HRpeak	12	481	1.70 (0.47, 2.92)	I^2^ =58% *p* = 0.007	*p* = 0.007
	Isocaloric consumption	Yes	7	267	1.80 (0.28, 3.31)	I^2^ = 65% *p* = 0.009	*p* = 0.02
		No	10	350	2.14 (0.99, 3.29)	I^2^ = 33% *p* = 0.14	*p* = 0.0003

**Table 4:** Egger’s test of the included studies.

Outcomes	*n*	Std. Err	t	*p* > |t|	95% CI	Interval
VO2peak	17	0.656	−1.87	0.081	−2.625	0.170
VE/VCO2 slope	9	2.093	0.18	0.862	−4.570	5.326
LVEF	10	0.340	2.84	0.022	0.182	1.746
HRrest	6	1.890	0.35	0.745	−4.590	5.905

## Data Availability

The data that support the findings of the study are available from the corresponding author, upon reasonable request.
